# High-dose clevudine impairs mitochondrial function and glucose-stimulated insulin secretion in INS-1E cells

**DOI:** 10.1186/1471-230X-12-4

**Published:** 2012-01-10

**Authors:** Yoon-Ok Jang, Xianglan Quan, Ranjan Das, Shanhua Xu, Choon-Hee Chung, Chan Mug Ahn, Soon-Koo Baik, In Deok Kong, Kyu-Sang Park, Moon Young Kim

**Affiliations:** 1 Department of Internal Medicine, Yonsei University, Wonju College of Medicine, Wonju, 220-701, Korea; 2 Department of Physiology, Yonsei University, Wonju College of Medicine, Wonju, 220-701, Korea; 3 Department of Basic Science, Yonsei University, Wonju College of Medicine, Wonju, 220-701, Korea; 4 Institute of Lifestyle Medicine, Yonsei University, Wonju College of Medicine, Wonju, 220-701, Korea

**Keywords:** clevudine, mitochondrial DNA, mitochondrial dysfunction, glucose-stimulated insulin secretion

## Abstract

**Background:**

Clevudine is a nucleoside analog reverse transcriptase inhibitor that exhibits potent antiviral activity against hepatitis B virus (HBV) without serious side effects. However, mitochondrial myopathy has been observed in patients with chronic HBV infection taking clevudine. Moreover, the development of diabetes was recently reported in patients receiving long-term treatment with clevudine. In this study, we investigated the effects of clevudine on mitochondrial function and insulin release in a rat clonal β-cell line, INS-1E.

**Methods:**

The mitochondrial DNA (mtDNA) copy number and the mRNA levels were measured by using quantitative PCR. MTT analysis, ATP/lactate measurements, and insulin assay were performed.

**Results:**

Both INS-1E cells and HepG2 cells, which originated from human hepatoma, showed dose-dependent decreases in mtDNA copy number and cytochrome c oxidase-1 (Cox-1) mRNA level following culture with clevudine (10 μM-1 mM) for 4 weeks. INS-1E cells treated with clevudine had reduced total mitochondrial activities, lower cytosolic ATP contents, enhanced lactate production, and more lipid accumulation. Insulin release in response to glucose application was markedly decreased in clevudine-treated INS-1E cells, which might be a consequence of mitochondrial dysfunction.

**Conclusions:**

Our data suggest that high-dose treatment with clevudine induces mitochondrial defects associated with mtDNA depletion and impairs glucose-stimulated insulin secretion in insulin-releasing cells. These findings partly explain the development of diabetes in patients receiving clevudine who might have a high susceptibility to mitochondrial toxicity.

## Background

Chronic infection with hepatitis B virus (HBV) frequently leads to serious liver disease such as cirrhosis, fulminant hepatic failure, and hepatocellular carcinoma [1]. Several antiviral drugs have been developed and prescribed for HBV infection. Commonly used antiviral therapies are nucleoside analog reverse transcriptase inhibitors (NRTIs) including entecavir, lamivudine, and telbivudine. NRTIs undergo intracellular and intramitochondrial phosphorylation into active triphosphates that are capable of inhibiting HIV reverse transcriptase (RT) [2]. However, these drugs have side effects such as lipodystrophy, neuropathy, myopathy, and liver steatosis, all of which are related to mitochondrial toxicity. *In vitro *and *in vivo *studies have shown that some NRTIs inhibit DNA polymerase-γ, a nuclear-encoded polymerase important for mitochondrial DNA (mtDNA) replication [3,4]. Depletion of mtDNA induced by NRTIs may attenuate mitochondrial oxidative phosphorylation, which could limit their clinical use.

Clevudine (1-(2-deoxy-2-fluoro-β-L-arabinofuranosyl) thymine) is an NRTI that exhibits potent and sustained antiviral activity against HBV with weaker effects on mitochondrial structure and function compared to those of other NRTIs [2,5]. However, long-term therapy for more than one year results in the development of considerable drug resistance and skeletal myopathy [6-9]. Muscle biopsies from patients with myopathy as a complication of clevudine treatment revealed severe necrosis with cytochrome c oxidase (COX)-negative ragged red fibers, the typical phenotype of mitochondrial myopathy [7,10]. Clevudine-induced myopathy developed in approximately 4-5% of patients and was usually reversible after discontinuation of clevudine [9].

It is well known that mitochondria play a critical role in nutrient-stimulated insulin secretion, as well as in insulin actions at target cells [11]. Recently, a patient who developed diabetes mellitus after clevudine treatment was reported [12]. We hypothesized that the mitochondrial dysfunction invoked by clevudine treatment could be a precipitating factor in diabetogenesis. Until now, the majority of *in vitro *studies for antiviral agent toxicities have been performed in different cell types, yielding conflicting results [13-15]. Insulin-secreting cells are highly specialized fuel sensors that maintain blood glucose level in the body by monitoring the ATP/ADP ratio, which is strictly regulated by mitochondrial oxidative phosphorylation. Thus, insulin-secreting cells are an appropriate model system for identification of mitochondrial toxicity and its functional consequences following antiviral therapy. In this study, we investigated the effects of clevudine exposure on mtDNA content, mitochondrial function, and metabolism-secretion coupling in insulin-releasing cells to elucidate the mechanism underlying the reversible diabetes observed in clevudine-treated patients.

## Methods

### Cell culture and drugs

Clevudine was purified from Revovir^® ^tablets (Bukwang Pharm. Co., Seoul, Korea). The amount of harvested clevudine was analyzed using HPLC (Agilent G1315B UV Diode array detector, AD, Santa Clara, CA, USA). A single peak with the expected amount of clevudine was measured based on the known weight of one tablet. INS-1E cell, a clonal pancreatic β-cell line received from Prof. Claes B. Wollheim, were cultured in complete medium composed of RPMI 1640 (Invitrogen, Carlsbad, CA, USA) supplemented with 10% fetal calf serum, 1 mM sodium pyruvate, 50 μM 2-mercaptoethanol, 2 mM glutamine, 10 mM HEPES, 100 units/ml penicillin, and 100 μg/ml streptomycin. HepG2 cells, a human hepatoma cell line, were grown in DMEM medium (Invitrogen) containing 5.6 mM glucose, 4 mM L-glutamine and 1 mM sodium pyruvate. For the following experiments, cells were cultured with or without clevudine for 4 weeks.

### Quantitative PCR

Total DNA or RNA was isolated and purified from INS-1E and HepG2 cells using DNeasy or RNeasy kits (Qiagen, Valencia, CA, USA), respectively. To obtain cDNA, reverse transcription (RT) was performed with oligo-dT (Applied Biosystems, Foster City, CA, USA) using reverse transcriptase (Promega, Madison, WI, USA). For PCR amplification, sequence-specific oligonucleotide primers for the genes of interest were designed (Bioneer, Daejeon, Korea) based on rat and human sequences in the GenBank database (Table 1). Quantitative real-time PCR using SYBR Green PCR Master Mix (Applied Biosystems) was performed in an ABI PRISM 7900HT Sequence Detection System (Applied Biosystems) according to the manufacturer's protocol. All amplifications were followed by melting curve analysis. The β-actin was used as the reference gene, and relative abundance of DNA or mRNA in clevudine-treated cells was normalized to that level in control cells calculated by using 2^-ΔΔCt ^method.

**Table 1:** Primers for quantitative PCR

Name		Primer sequence	Accession code	product size
rat β-actin	**+**	ATGGTGGGTATGGGTCAGAA	NM_031144.2	100 bp
	**-**	TCCATATCGTCCCAGTTGGT		
rat Cox1	**+**	GGAGCAGTATTCGCCATCAT	NC_001665	90 bp
	**-**	GTGGGCTTTTGCTCATGTGT		
rat PGC1a	**+**	GGCACATCTGTTCTTCCACA	NM_031347.1	110 bp
	**-**	TTCCTGGTCTTGGAGCTGTT		
rat NRF1	**+**	GGACAGCAAGCCATTGTTCT	NM_001100708.1	98 bp
	**-**	TACTTGCGCACCACATTCTC		
rat Tfam	**+**	GCTGAGTGGAAGGTGTACAAAG	NM_031326.1	85 bp
	**-**	CTTCCTTCTCTAAGCCCATCAG		
rat SDH	**+**	TCTTTCCTACCCGCTCACAT	NM_130428.1	90 bp
	**-**	AATGCCATCTCCAGTTGTCC		
human β-actin	**+**	AAGTTCACAATGTGGCCGAG	NM_001101.3	98 bp
	**-**	ATGGCAAGGGACTTCCTGTA		
human Cox1	**+**	CACACTCCACGGAAGCAATA	NC_012920.1	82 bp
	**-**	GCCACCTACGGTGAAAAGAA		

### MTT assay

3-(4,5-dimethylhioazol-2-yl)-2,5-diphenyltetrazolium bromide (MTT) was purchased from Sigma (St. Louis, MO, USA). INS-1E cells seeded onto a 96-well plate (5 × 10^4 ^cells/well) were incubated with MTT (50 μg/well) for 2 hrs, and then the medium was discarded and replaced with dimethylsulfoxide (100 μl/well). The absorbance of each well was measured at 570 nm using an enzyme-linked immunosorbent assay (ELISA) reader, after background subtraction at 650 nm.

### Cytochrome c oxidase (COX) activity measurement

INS-1E cells were harvested and incubated with isosmotic medium [16] containing 0.2% triton X-100 at 30°C for 2 min. Enzymatic activity of COX was measured spectrophotometrically at 550 nm based on previous reports [16,17].

### ATP and lactate measurements

INS-1E cells seeded onto 24-well plates (3 × 10^5 ^cells/well) were preincubated with glucose-free medium for 2 hrs prior to incubation with KRBH solution (135 mM NaCl, 3.6 mM KCl, 2 mM NaHCO_3_, 0.5 mM NaH_2_PO_4_, 0.5 mM MgSO_4_, 1.5 mM CaCl_2_, 10 mM HEPES, pH 7.4) containing 2.8 mM glucose for 30 min. The cells were then stimulated for 15 min with KRBH buffer at a low (2.8 mM) or high (16.7 mM) glucose concentration. The ATP content in the cell lysate (Roche HS-II Biolumniscence kit, Mannheim, Germany) and the lactate level in the cell supernatant (Biovision #K607-100, Mountain View, CA, USA) were measured as described previously [18]. Measurement of the protein concentration in cell lysates was performed using the Bradford assay.

### Oil red staining

INS-1E cells on coverslip were treated with bovine serum albumin (BSA) or oleate, a monounsaturated fatty acid for 24 hours. After fixation with 10% formalin, cells were washed with 60% isopropanol and dried at room temperature. Cells were incubated with Oil Red O (Sigma, St. Louis, MO, USA) for 10 min, and then counterstained with hematoxylin.

### Insulin measurement

INS-1E cells were seeded and cultured as for ATP and lactate measurement. For insulin measurement, 0.1% BSA was included in the KRBH solution, and the cells were stimulated with low or high concentrations of glucose for 30 min, as described previously [18]. Insulin levels in supernatant and cell extracts were measured using a rat insulin enzyme immunoassay kit (Shibayagi Co., Gunma, Japan).

### Data analysis

All data are presented as mean ± SEM, and the statistical significance was determined using One-way ANOVA or Student's t test.

## Results

### Effects of clevudine on mtDNA copy number and mRNA levels of mtDNA encoded genes

**Figure 1:**
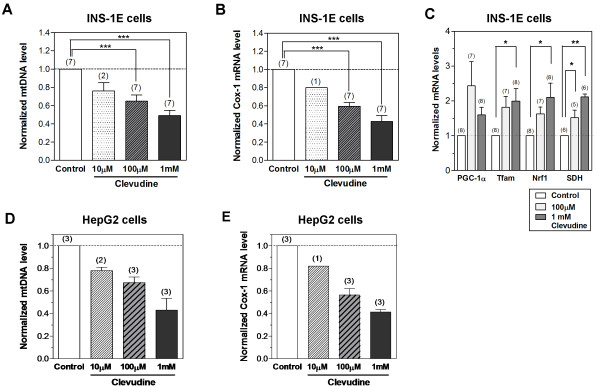
**Effects of clevudine on mitochondrial DNA copy number and mRNA levels of mitochondria-related genes**. The mitochondrial DNA copy number and the mRNA levels of mtDNA-encoded or nuclear DNA-encoded genes were measured using quantitative PCR. Clevudine reduced the expressions of mtDNA (A & D) and cytochrome *c *oxidase-1 (*Cox-1*) mRNA (B & E) in the rat clonal β-cell line INS-1E and in the human hepatoma cell line HepG2 in a dose-dependent manner. Nuclear-encoded genes related to mitochondrial biogenesis such as PPAR-γ coactivator 1α (*PGC-1α*), mitochondrial transcription factor A (*Tfam*), and nuclear respiratory factor 1 (*NRF1*) were upregulated by clevudine treatment (C). Succinate dehydrogenase (SDH) was also upregulated by clevudine (C). Data presented are means ± SEM. The number of experiments is shown in parenthesis. *, **, and *** denote p < 0.05, p < 0.01, and p < 0.001, respectively.

INS-1E cells were cultured with different concentrations of clevudine for 4 weeks and the *in vitro *effects on mtDNA replication and translation were measured. Treatment with clevudine (10 μM to 1 mM) reduced the mtDNA copy number in a dose-dependent manner (Figure 1A). Two weeks treatment with clevudine induced 39% reduction of mtDNA level (n = 3), which was smaller than four weeks treatment (51%). The mRNA levels of mtDNA-encoded *Cox-1 *were also dose-dependently attenuated by clevudine (Figure 1B). Interestingly, we observed upregulation of PPAR- γ coactivator 1α (*PGC-1α*), mitochondrial transcription factor A (*Tfam*), and nuclear respiratory factor 1 (*NRF1*) in clevudine-treated INS-1E cells. Upregulation of these transcription factors could be a nuclear response to mitochondrial dysfunction [3]. Succinate dehydrogenase (SDH), a nuclear-encoded mitochondrial enzyme, was also upregulated by clevudine-treatment (Figure 1C). We next examined the effects of clevudine on the levels of mtDNA and RNA in the human hepatoma cell line HepG2, major target cells of insulin action. Clevudine showed suppressive effects on mtDNA replication and transcription in HepG2 cells, similar to the effect in INS-1E cells (Figures 1D and 1E).

### Mitochondrial dysfunction induced by clevudine

**Figure 2:**
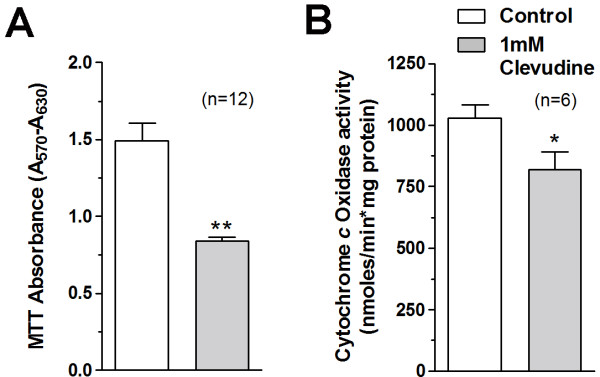
**High-dose clevudine decreased the mitochondrial activities in INS-1E cells**. (A) Clevudine reduced the MTT signal, which reflects total mitochondrial activity within each well. (B) Clevudine decreased the enzymatic activity of cytochrome c oxidase (COX). Data are presented as means ± SEM. The number of experiments is shown in parenthesis. * and ** denote p < 0.05 and p < 0.01, respectively.

The amount of formazan reaction product formed in the MTT assay reflects the total mitochondrial enzymatic activity in each well. INS-1E cells treated with or without clevudine for 4 weeks were seeded 48 hrs before the MTT assay. Exposure to clevudine decreased the MTT absorbance (71% by 100 μM and 56% by 1 mM, Figure 2A). However, we observed that there was no significant difference in protein amount between control cells (59 ± 3 μg; n = 17) and cells incubated with 100 μM (62 ± 4 μg; n = 17) or 1 mM clevudine (59 ± 3 μg; n = 17) for 48 hours after seeding (3 × 10^5 ^cells). This result implies that reduction of MTT signal by clevudine might be resulted from decreased mitochondrial reducing capacity. To demonstrate the functional significance of decreased *Cox1 *mRNA, we performed the enzymatic activity measurement of cytochrome c oxidase (Cox) based on previous reports [16,17]. We observed the reduced Cox activity of clevudine (1 mM)-treated cells compared to that of control INS-1E cells (Figure 2B).

**Figure 3:**
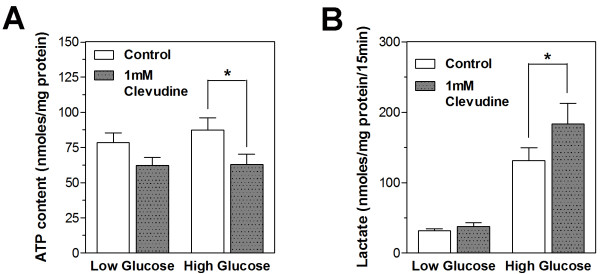
**High-dose clevudine decreased ATP level and increased lactate production in INS-1E cells**. Cytosolic ATP level was reduced (A), but glucose-stimulated lactate production was enhanced (B) in 1 mM clevudine-treated cells. Data are presented as means ± SEM. * denotes p < 0.05.

We measured the cellular contents of ATP in control and clevudine-treated INS-1E cells using a bioluminescence method after incubation with low (2.8 mM) or high (16.7 mM) concentrations of glucose for 15 min. As shown in Figure 3A, cells cultured with 1 mM clevudine had lower cytosolic ATP level in both low and high glucose conditions than control cells. Lactate production from INS-1E cells was markedly elevated by incubation with glucose for 15 min (Figure 3B). The glucose-induced lactate production was increased in cells treated with 1 mM clevudine compared to that in control cells (Figure 3B).

**Figure 4:**
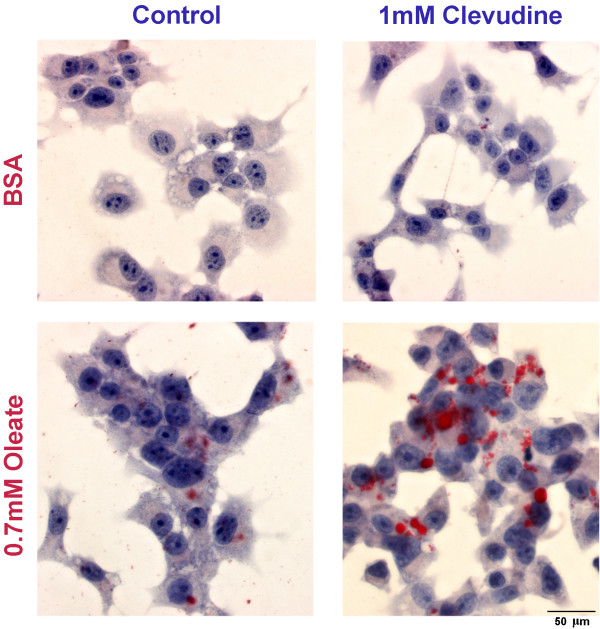
**Effects of clevudine on lipid accumulation in INS-1E cells**. INS-1E cells on coverslip were treated with 0.7 mM oleate or bovine serum albumin (BSA) for 24 hours, and stained Oil Red O with hematoxylin. Clevudine (1 mM)-treated cells showed a pronounced lipid accumulation (red droplets) upon oleate incubation, which was much less in control cells.

One of the metabolic consequences of mitochondrial dysfunction is an impairment of fatty acid oxidation, thus leading to lipid accumulation [19,20]. To detect the lipid droplet in cytosol, we performed Oil-red O staining to control and clevudine-treated cells. Without exogenous fatty acid loading, there was no significant difference between two groups. When we incubated cells with a mono-unsaturated fatty acid, 0.7 mM oleate, clevudine-treated cells showed a pronounced lipid accumulation, which was much less in control cells (Figure 4).

### Inhibition of glucose-stimulated insulin secretion by clevudine

**Figure 5:**
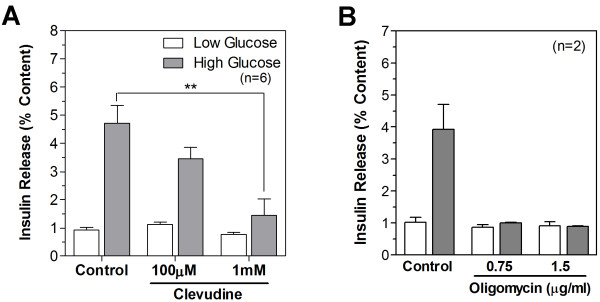
**Clevudine inhibited glucose-stimulated insulin secretion**. Released insulin and cellular insulin content were measured using an enzymatic immunoassay after incubation with Krebs buffer containing 2.8 mM or 16.7 mM glucose for 15 min. Glucose-stimulated insulin secretion was impaired by clevudine incubation (A), as well as by inhibiting mitochondrial ATP synthase with oligomycin (B). Insulin release was normalized to the insulin content. Data presented are mean ± SEM. ** denotes p < 0.01.

To identify whether clevudine-induced mitochondrial dysfunction affects insulin secretory activity, we measured the released and cellular contents of insulin via an enzyme immunoassay. The cellular insulin contents were not significantly different between control INS-1E cells (816 ± 167 ng/well) and cells treated with clevudine (1 mM) for 4 weeks (769 ± 170 ng/well). After incubation for 30 min with low (2.8 mM) or high (16.7 mM) concentrations of glucose, the released insulin was normalized to the cellular content and expressed as a percentage of the content released. We observed that high concentration glucose stimulated the release of insulin by 5.1-fold in control cells but by only 3.1-fold and 1.9-fold in cells treated with 100 μM and 1 mM clevudine for 4 weeks, respectively (Figure 3). There was no difference in % insulin releases induced by low concentration glucose between the control and clevudine-treated groups (Figure 5A). We observed that the glucose-stimulated insulin secretion was completely abolished by the treatment with oligomycin (0.75 μg/ml), a mitochondrial ATP synthase inhibitor (Figure 5B). This result demonstrates the cause-effect relationship between mitochondrial dysfunction and impaired insulin secretion.

## Discussion

In pancreatic β-cells, mitochondria are of particular importance in the regulation of insulin secretion because they produce ATP as well as other coupling factors which link nutrient metabolism and insulin exocytosis [11]. mtDNA-depleted β-cell lines show complete absence of nutrient-stimulated insulin secretion [21]. Patients with mtDNA mutations develop diabetes, accounting for up to 1% of the total number of diabetic patients [22]. Moreover, postmortem islets from type 2 diabetes patients display functional deterioration of mitochondria [23]. Therefore, factors that disturb the mitochondrial function in pancreatic β-cells might affect metabolism-secretion coupling and diabetogenesis.

The present study showed that the effective anti-HBV agent clevudine has a negative effect on the copy number and transcription of mtDNA in insulin-releasing cells and hepatoma cells. The reduced expressions of mtDNA-encoded proteins lead to attenuation of mitochondrial function. In insulin-releasing cells, clevudine-induced mitochondrial dysfunction can elicit defective insulin secretion in response to substrates for mitochondrial metabolism. To our knowledge, this is the first demonstration that an antiviral agent can impair nutrient-stimulated insulin secretion as a result of mitochondrial dysfunction. Because of their high dependency on mitochondrial function in metabolism-secretion coupling, insulin-secreting cells provide a useful model to investigate the functional consequences of drug-induced mitochondrial toxicity.

NRTIs are widely used to treat various viral diseases such as acquired immunodeficiency syndrome (AIDS) and hepatitis B [24]. However, *in vitro *studies showed that NRTIs can alter mtDNA content by inhibiting DNA polymerase-γ [25]. Moreover, myopathy accompanied by mtDNA depletion has been reported in NRTI-treated patients [4]. Clevudine treatment has also been associated with the development of mitochondrial complications. In contrast to early studies [2], depletion of mtDNA in skeletal muscle has been observed in patients treated with clevudine [7,26]. Typical histological features of mitochondrial myopathy and abnormal mitochondrial morphology were displayed in tissues from patients with increased lactate dehydrogenase and lactate levels [8,10]. Although the incidence of clevudine-induced myopathy was reported to be low (~5%) [9], a substantial proportion (~14.5%) of clevudine-treated patients have been found to experience symptoms, signs, and laboratory abnormalities relevant to clevudine-induced myopathy [27].

To directly confirm the effects of clevudine on mitochondrial function, we cultured cells with medium containing different concentrations of clevudine for 4 weeks. Clevudine markedly decreased the MTT signal without significant changes in cellular protein implying the diminished enzyme activities for reduction of MTT. Since MTT assay is not specific to evaluate mitochondrial function, measurement of oxygen consumption rate or citrate synthase activity could provide more concrete evidence to prove the mitochondrial defects. Consistent with mtDNA depletion, COX activity and cellular ATP content were reduced. Decreased mitochondrial fatty acid oxidation could induce triglyceride accumulation [19]. To avoid lipotoxic effects of palmitate in insulin-secreting cells [28], we loaded unsaturated fatty acid oleate for 24 hours, which elicited a marked increase of lipid accretion within clevudine-treated cells. The inhibitory effect of clevudine on insulin secretion was more sensitive than the effect on ATP level. We can speculate that the treatment of 100 μM clevudine elicited significant reduction of ATP/ADP ratio which is the main signal for closing ATP-sensitive K^+ ^channel and insulin exocytosis.

We also observed some compensatory responses to reduced mtDNA copy number and its functional consequences. First, PGC-1α and its downstream transcriptional factors, NRF-1 and Tfam, were upregulated by clevudine. Second, nuclear DNA-encoded succinate dehydrogenase was also upregulated, which has already been observed in muscle of patients suffering from clevudine-induced myopathy [10]. Third, lactate production was modestly increased in association with diminished ATP content. Pancreatic β-cells and clonal β-cell lines are known to have very low lactate dehydrogenase levels, which contribute to their dependency on mitochondrial function. The increase in lactate production observed in our study also demonstrates that clevudine imposes selective defects on mitochondria rather than overall cytotoxicity.

In our study, mtDNA copy number in clevudine (1 mM)-treated cells was decreased to half of that in control. It has been reported, however, that to evoke mitochondrial dysfunction mtDNA level should fall below 60% which was named as 'phenotypic threshold' [29]. This can be explained by genetic and functional complementation at the levels of transcription, translation, enzyme activity and cell activity. Several investigators showed that NRTI such as zidovudine and stavudine can also induce mitochondrial dysfunction independent from lack of mtDNA [20,30]. Thus, we cannot exclude the possibility that clevudine could be involved in multiple site of inhibition of mitochondrial function in addition to the effects of mtDNA depletion.

Niu et al. [31] suggested that the intracellular level of the triphosphate form of clevudine in cells exposed to 1 μM extracellular clevudine approximates the plasma level in patients receiving a 30 mg dose. Our results indicated that impairments in mitochondrial function and insulin secretion are elicited only by high concentrations of clevudine (> 100 μM). This means that clevudine would minimally affect mitochondrial function within the therapeutic concentration range. It is noteworthy, however, that mutations or polymorphisms of DNA polymerase-γ were identified in NRTI-treated patients with mitochondrial complications [32]. This suggests that genetic alterations in DNA polymerase-γ are not normally deleterious, but that certain conditions such as NRTI treatment may push mitochondrial activity below the clinical threshold, causing pathogenic dysfunction [33]. Differences in genetic susceptibility to mitochondrial toxicity could be one explanation for why a limited proportion of patients receiving clevudine have complications including myopathy.

Clevudine-induced depletion of mtDNA is not restricted to insulin-secreting cells but is also observed in cultured hepatoma cells or muscle tissue from patients [7,26]. Mitochondrial dysfunction in insulin target tissues such as liver and muscle could result in insulin resistance and diabetes [34]. In addition to defects in insulin secretion, decreased sensitivity in insulin target cells can also participate in diabetogenesis in patients receiving clevudine who might have a high susceptibility to mitochondrial toxicity. Interestingly, several reports have shown that NRTI induces intramitochondrial pyrimidine deficiency which may aggravate mtDNA depletion and mitochondrial dysfunction [35,36]. They also discovered that uridine supplementation attenuates steatohepatitis or mitochondrial myopathy induced by NRTI. Further studies concerning the effects of NRTIs on mitochondrial function in different cell types may help us understanding these intractable complications and develop novel antiviral agents.

## Conclusions

In summary, clevudine, used as an antiviral agent against chronic hepatitis B, significantly decreased the mtDNA copy number at higher doses compared to therapeutic concentration. Mitochondrial dysfunction due to depleted mtDNA and defective ATP synthesis in insulin-releasing cells, consequently led to the impairment of glucose-stimulated insulin secretion. Clevudine-induced mitochondrial dysfunction may contribute to diabetogenesis among clevudine-treated patients who might be more susceptible to mitochondrial toxicity.

## Competing interests

The authors declare that they have no competing interests.

## Authors' contributions

KSP and MYK designed the project. YOJ, XQ, RD, and SX performed the experiments. YOJ, KSP, and MYK wrote the manuscript. CMA prepared a purified clevudine. CHC, CMA, SKB, and IDK contributed to the discussion of the data and the revision of the manuscript. All readers read and approved the final manuscript.

## Pre-publication history

The pre-publication history for this paper can be accessed here:

http://www.biomedcentral.com/1471-230X/12/4/prepub

## References

[B1] Sorrell MF Belongia EA Costa J Gareen IF Grem JL Inadomi JM Kern ER McHugh JA Petersen GM Rein MF National Institutes of Health Consensus Development Conference Statement: management of hepatitis B Ann Intern Med 2009 150 2 104 110 19124811 10.7326/0003-4819-150-2-200901200-00100

[B2] Balakrishna Pai S Liu SH Zhu YL Chu CK Cheng YC Inhibition of hepatitis B virus by a novel L-nucleoside, 2'-fluoro-5-methyl-beta-L-arabinofuranosyl uracil Antimicrobial agents and chemotherapy 1996 40 2 380 386 8834884 10.1128/aac.40.2.380 PMC163120

[B3] Mallon PW Unemori P Sedwell R Morey A Rafferty M Williams K Chisholm D Samaras K Emery S Kelleher A In vivo, nucleoside reverse-transcriptase inhibitors alter expression of both mitochondrial and lipid metabolism genes in the absence of depletion of mitochondrial DNA J Infect Dis 2005 191 10 1686 1696 10.1086/429697 15838796

[B4] Lewis W Day BJ Copeland WC Mitochondrial toxicity of NRTI antiviral drugs: an integrated cellular perspective Nat Rev Drug Discov 2003 2 10 812 822 10.1038/nrd1201 14526384

[B5] Yao GQ Liu SH Chou E Kukhanova M Chu CK Cheng YC Inhibition of Epstein-Barr virus replication by a novel L-nucleoside, 2'-fluoro-5-methyl-beta-L-arabinofuranosyluracil Biochem Pharmacol 1996 51 7 941 947 10.1016/0006-2952(96)00049-4 8651944

[B6] Kwon SY Park YK Ahn SH Cho ES Choe WH Lee CH Kim BK Ko SY Choi HS Park ES Identification and characterization of clevudine-resistant mutants of hepatitis B virus isolated from chronic hepatitis B patients J Virol 2010 84 9 4494 4503 10.1128/JVI.02066-09 20164224 PMC2863790

[B7] Seok JI Lee DK Lee CH Park MS Kim SY Kim HS Jo HY Kim DS Long-term therapy with clevudine for chronic hepatitis B can be associated with myopathy characterized by depletion of mitochondrial DNA Hepatology 2009 49 6 2080 2086 10.1002/hep.22959 19333909

[B8] Tak WY Park SY Jung MK Jeon SW Cho CM Kweon YO Kim SK Choi YH Mitochondrial myopathy caused by clevudine therapy in chronic hepatitis B patients Hepatol Res 2009 39 9 944 947 10.1111/j.1872-034X.2009.00515.x 19712273

[B9] Jang JH Kim JW Jeong SH Myung HJ Kim HS Park YS Lee SH Hwang JH Kim N Lee DH Clevudine for chronic hepatitis B: antiviral response, predictors of response, and development of myopathy J Viral Hepat 2011 18 2 84 90 10.1111/j.1365-2893.2010.01281.x 20196804

[B10] Tak WY Park SY Cho CM Jung MK Jeon SW Kweon YO Park JY Sohn YK Clinical, biochemical, and pathological characteristics of clevudine-associated myopathy J Hepatol 2010 53 2 261 266 10.1016/j.jhep.2010.03.006 20466447

[B11] Wiederkehr A Wollheim CB Minireview: implication of mitochondria in insulin secretion and action Endocrinology 2006 147 6 2643 2649 10.1210/en.2006-0057 16556766

[B12] Kim GW Lee MY Kim SY Kim JH Lee JH Chung CH Clevudine induced diabetes mellitus in a patient with chronic hepatitis B Korean J Med 2010 79 5 569 572

[B13] Nolan D Hammond E Martin A Taylor L Herrmann S McKinnon E Metcalf C Latham B Mallal S Mitochondrial DNA depletion and morphologic changes in adipocytes associated with nucleoside reverse transcriptase inhibitor therapy AIDS 2003 17 9 1329 1338 10.1097/00002030-200306130-00007 12799554

[B14] Miura T Goto M Hosoya N Odawara T Kitamura Y Nakamura T Iwamoto A Depletion of mitochondrial DNA in HIV-1-infected patients and its amelioration by antiretroviral therapy J Med Virol 2003 70 4 497 505 10.1002/jmv.10423 12794710

[B15] Stankov MV Lucke T Das AM Schmidt RE Behrens GM Relationship of mitochondrial DNA depletion and respiratory chain activity in preadipocytes treated with nucleoside reverse transcriptase inhibitors Antivir Ther 2007 12 2 205 216 17503663

[B16] Barrientos A In vivo and in organello assessment of OXPHOS activities Methods 2002 26 4 307 316 10.1016/S1046-2023(02)00036-1 12054921

[B17] Miro O Cardellach F Barrientos A Casademont J Rotig A Rustin P Cytochrome c oxidase assay in minute amounts of human skeletal muscle using single wavelength spectrophotometers Journal of neuroscience methods 1998 80 1 107 111 10.1016/S0165-0270(97)00204-5 9606056

[B18] Park KS Wiederkehr A Kirkpatrick C Mattenberger Y Martinou JC Marchetti P Demaurex N Wollheim CB Selective actions of mitochondrial fission/fusion genes on metabolism-secretion coupling in insulin-releasing cells J Biol Chem 2008 283 48 33347 33356 10.1074/jbc.M806251200 18832378 PMC2662262

[B19] Fromenty B Pessayre D Inhibition of mitochondrial beta-oxidation as a mechanism of hepatotoxicity Pharmacology & therapeutics 1995 67 1 101 154 10.1016/0163-7258(95)00012-6 7494860

[B20] Igoudjil A Massart J Begriche K Descatoire V Robin MA Fromenty B High concentrations of stavudine impair fatty acid oxidation without depleting mitochondrial DNA in cultured rat hepatocytes Toxicology in vitro: an international journal published in association with BIBRA 2008 22 4 887 898 10.1016/j.tiv.2008.01.011 18299183

[B21] Kennedy ED Maechler P Wollheim CB Effects of depletion of mitochondrial DNA in metabolism secretion coupling in INS-1 cells Diabetes 1998 47 3 374 380 10.2337/diabetes.47.3.374 9519742

[B22] Maassen JA Janssen GM t Hart LM Molecular mechanisms of mitochondrial diabetes (MIDD) Ann Med 2005 37 3 213 221 10.1080/07853890510007188 16019720

[B23] Deng S Vatamaniuk M Huang X Doliba N Lian MM Frank A Velidedeoglu E Desai NM Koeberlein B Wolf B Structural and functional abnormalities in the islets isolated from type 2 diabetic subjects Diabetes 2004 53 3 624 632 10.2337/diabetes.53.3.624 14988246

[B24] Pinti M Salomoni P Cossarizza A Anti-HIV drugs and the mitochondria Biochim Biophys Acta 2006 1757 5-6 700 707 10.1016/j.bbabio.2006.05.001 16782042

[B25] Lim SE Copeland WC Differential incorporation and removal of antiviral deoxynucleotides by human DNA polymerase gamma J Biol Chem 2001 276 26 23616 23623 10.1074/jbc.M101114200 11319228

[B26] Fleischer RD Lok AS Myopathy and neuropathy associated with nucleos(t)ide analog therapy for hepatitis B J Hepatol 2009 51 4 787 791 10.1016/j.jhep.2009.06.011 19665816

[B27] Kim HJ Park DI Park JH Cho YK Sohn CI Jeon WK Kim BI Comparison between clevudine and entecavir treatment for antiviral-naive patients with chronic hepatitis B Liver Int 2010 30 6 834 840 20408946 10.1111/j.1478-3231.2010.02245.x

[B28] Cunha DA Hekerman P Ladriere L Bazarra-Castro A Ortis F Wakeham MC Moore F Rasschaert J Cardozo AK Bellomo E Initiation and execution of lipotoxic ER stress in pancreatic beta-cells Journal of cell science 2008 121 Pt 14 2308 2318 18559892 10.1242/jcs.026062 PMC3675788

[B29] Rossignol R Faustin B Rocher C Malgat M Mazat JP Letellier T Mitochondrial threshold effects The Biochemical journal 2003 370 Pt 3 751 762 12467494 10.1042/BJ20021594 PMC1223225

[B30] Pan-Zhou XR Cui L Zhou XJ Sommadossi JP Darley-Usmar VM Differential effects of antiretroviral nucleoside analogs on mitochondrial function in HepG2 cells Antimicrobial agents and chemotherapy 2000 44 3 496 503 10.1128/AAC.44.3.496-503.2000 10681309 PMC89717

[B31] Niu C Murakami E Furman PA Clevudine is efficiently phosphorylated to the active triphosphate form in primary human hepatocytes Antivir Ther 2008 13 2 263 269 18505177

[B32] Yamanaka H Gatanaga H Kosalaraksa P Matsuoka-Aizawa S Takahashi T Kimura S Oka S Novel mutation of human DNA polymerase gamma associated with mitochondrial toxicity induced by anti-HIV treatment J Infect Dis 2007 195 10 1419 1425 10.1086/513872 17436221

[B33] Chan SS Copeland WC DNA polymerase gamma and mitochondrial disease: understanding the consequence of POLG mutations Biochim Biophys Acta 2009 1787 5 312 319 10.1016/j.bbabio.2008.10.007 19010300 PMC2742478

[B34] Wang CH Wang CC Wei YH Mitochondrial dysfunction in insulin insensitivity: implication of mitochondrial role in type 2 diabetes Ann N Y Acad Sci 2010 1201 157 165 10.1111/j.1749-6632.2010.05625.x 20649552

[B35] Lebrecht D Vargas-Infante YA Setzer B Kirschner J Walker UA Uridine supplementation antagonizes zalcitabine-induced microvesicular steatohepatitis in mice Hepatology 2007 45 1 72 79 10.1002/hep.21490 17187420

[B36] Lebrecht D Deveaud C Beauvoit B Bonnet J Kirschner J Walker UA Uridine supplementation antagonizes zidovudine-induced mitochondrial myopathy and hyperlactatemia in mice Arthritis and rheumatism 2008 58 1 318 326 10.1002/art.23235 18163507

